# Vitrification of Rhesus Macaque Mesenchymal Stem Cells and the Effects on Global Gene Expression

**DOI:** 10.1155/2017/3893691

**Published:** 2017-10-24

**Authors:** Xufeng Fu, Yaping Yan, Shanshan Li, Junfeng Wang, Bin Jiang, Hong Wang, Yanchao Duan, Tao Tan, Fei Gao, Desheng Gong, Yuyu Niu, Weizhi Ji, Bingrong Zheng, Wei Si

**Affiliations:** ^1^Yunnan Key Laboratory of Primate Biomedical Research, Institute of Primate Translational Medicine, Kunming University of Science and Technology, Kunming 650500, China; ^2^School of Medicine, Yunnan University, Kunming 650091, China; ^3^Key Laboratory of Fertility Preservation and Maintenance of Ministry of Education, Ningxia Medical University, Yinchuan 750004, China; ^4^Department of Hepatic and Bile Duct Surgery, The First People's Hospital of Yunnan Province, Kunming 650032, China; ^5^Yunnan Provincial Academy of Science and Technology, Kunming 650500, China; ^6^Kunming Ennovate Institute of Bioscience, Kunming 650500, China; ^7^Agricultural Genomics Institute at Shenzhen, Chinese Academy of Agricultural Sciences, Shenzhen 518120, China

## Abstract

Mesenchymal stem cells (MSCs) are one of the most promising adult stem cells for clinical application in a cell therapy. The development of large-scale cryopreservation techniques, such as vitrification, for MSCs is a prerequisite for clinical therapies. Dimethyl sulfoxide (DMSO) and ethylene glycol (EG) are two types of cryoprotectants widely used for cell vitrification. However, the effects of DMSO and EG on the biological characteristics and transcriptome profiles of MSCs after cryopreservation remain unknown. In the present study, the viability, immunophenotype of cell surface markers, proliferation, differentiation potency, and global gene expression of rhesus macaque bone marrow-derived MSCs vitrified using DMSO and EG were studied. The results showed that vitrification did not affect the morphology, surface markers, and differentiation of the MSCs, and compared to DMSO, EG better protected cell viability and proliferation. Most importantly, vitrification resulted in changes in a large number of transcripts of MSCs either preserved using DMSO or EG. This report is the first to examine the effects of DMSO and EG on global gene expression in stem cells. These results will be beneficial to understanding the biological process involved in MSC vitrification and will contribute to improving cryopreservation protocols that maintain transcriptomic identity with high cryosurvival for preclinical research and clinical long-term storage.

## 1. Introduction

Mesenchymal stem cells are spindle-shaped fibroblast-like adult stem cells that are easy to isolate, culture, and expand *in vitro*. Mesenchymal stem cells can be differentiated into various cell types *in vitro* and *in vivo* under appropriate conditions, reflecting their multipotent capacity [1]. In addition to direct conversion into differentiated cells for tissue regeneration, the therapeutic mechanisms of MSCs also include the immunosuppression and secretion of growth factors and the promotion of endogenous regenerative processes. Moreover, there are fewer ethical issues associated with MSCs than embryonic stem cells for clinical applications [2]. Therefore, MSCs can be used in the treatment of a variety of clinical conditions and have been regarded as one of the most promising adult stem cells for clinical applications in cell therapy and regenerative medicine. The success of regenerative treatment with MSCs in clinical trials requires a large number of cells. For example, approximately 10^6^ MSCs per kilogram of body weight and 10^8^ MSCs for one patient were infused in cell therapy. However, the long-term cultivation of MSCs can result in the loss of progenitor properties and generate malignant transformation due to changes in gene expression related to cell differentiation [3, 4], alterations of cell and mitochondrial morphology, the generation of reactive oxygen species, and the decrease in antioxidant capacities [5]. Therefore, the development of an optimal cryopreservation technique is a prerequisite for large-scale MSCs and storage for clinical therapies [6].

Cryopreservation provides a practical and effective method for maintaining the potency of stem cells with low cost and less labor. Traditionally, MSCs are cryopreserved at a slow cooling rate using DMSO as a cryoprotectant. Cells in freezing medium containing 5–10% DMSO are packed into cryovials and frozen in a computer programmed freezer at a cooling rate of −1°C/min to −80°C prior to freezing in liquid nitrogen for storage [7, 8]. Vitrification is the process of cryopreservation using high concentrations of cryoprotectants and rapid cooling rates, which promptly transform the vitrification solution into a glass-like state without ice formation during cooling [9, 10]. Vitrification has gained popularity in recent years, reflecting cost-effective and time-saving features, and this technique has successfully been used for the cryopreservation of embryos, oocytes, embryonic stem cells, tissues, and organs [11, 12]. Dimethyl sulfoxide is widely used for cell cryopreservation for both slow freezing and vitrification because of its superior membrane-penetrating and water displacement properties. Previous studies have reported that the long-term cryostorage of MSCs in 10% DMSO did not influence the proliferative characteristics, senescence, karyotype, and plasticity of MSCs [13]. However, other studies have revealed the negative effects of DMSO on cells. DMSO induced apoptosis in cells through caspase activation and plasma membrane pore formation, altered ATP synthesis, mtDNA copy, and mitochondrial function [14, 15]. Furthermore, adverse reactions, including nausea, headache, hypotension, hypertension, diarrhea, and abdominal cramps, have been reported in patients infused with cryopreserved stem cells using DMSO as a cryoprotectant [16, 17]. Recent studies have revealed that traditional slow freezing could result in apoptotic cell death and cell cycle regulator gene expression of MSCs [18, 19]. Alternatively, another penetrating cryoprotectant EG has been used in the slow freezing and vitrification of multiple cell types, including sperm, oocytes, ovarian follicles, embryos, and MSCs [20–23], and EG has been suggested as a more appropriate penetrating cryoprotectant than DMSO, reflecting its lower polarity and molecular mass, reduced toxicity, and higher permeability coefficient [24]. The cryoprotective roles and additional effects of the two penetrating cryoprotectants on global gene expression, however, have not been studied and compared for the cryopreservation of MSCs.

The rhesus macaque is one of the most widely used laboratory animals in biomedical research because of its genetic, physiological, behavioral, and neurological similarities to humans, and the macaque provides excellent translational validity in preclinical studies [25]. The present study was aimed to compare the cryoprotective effects of DMSO and EG on vitrification of rhesus macaque MSCs and improve the cryosurvival of MSCs. In addition, the further influence of vitrification with DMSO and EG on the global gene expression of MSCs was examined to facilitate future applications of MSCs in regenerative medicine.

## 2. Materials and Methods

### 2.1. Animals

Three male rhesus macaques (2 years old) were used as bone marrow donors. The procedure for bone marrow retrieval was approved through the Institutional Animal Care and Use Committee of Kunming University of Science and Technology and performed in accordance with the Guide for the Care and Use of Laboratory Animals.

### 2.2. Preparation and Culture of MSCs

The bone marrow-derived MSCs were isolated from the tibias of the young rhesus macaques. The muscular tissues on tibias were carefully removed. The ends of the bones were cut, and bone marrow was aseptically flushed ten times using a sterile syringe containing 10 mL of Dulbecco's modified Eagle's medium (DMEM) (Gibco BRL, Grand Island, NY, USA) supplemented with 10% (*v*/*v*) fetal bovine serum (FBS) (Gibco) and 1% (*v*/*v*) penicillin/streptomycin (Gibco). The cell suspension was subsequently centrifuged at 500*g* for 5 minutes, and the supernatant was discarded. Next, the marrow cells were mechanically dispersed into a single-cell suspension and seeded onto 10 cm plastic dishes at a density of 1 × 10^6^ cells/ml. The cells were cultured in DMEM medium supplemented with 10% FBS at 37°C in an incubator with a humidified atmosphere of 5% CO_2_. The nonadherent cells were removed, and the medium was refreshed every 48 hours. Ten days later, the primary cell culture (passage 0) was passaged at 80% confluency using 0.25% trypsin (Gibco). The cells were resuspended in culture medium at a dilution ratio of 1 : 3 and expanded on a new plastic petri dish to passage 1. The morphology, surface markers, and differentiation potency of MSCs were identified at passage 3. The MSCs were expanded up to passage 5 and subsequently subjected to vitrification and global gene expression examination as described below.

### 2.3. Flow Cytometry Analysis of the Immunophenotype Surface Markers of MSCs

The expression of surface markers of MSCs was examined using flow cytometry analysis (BD Biosciences, San Jose, CA). All antibodies were purchased from BD Biosciences. Approximately, 5 × 10^5^ MSCs were collected and washed with 500 *μ*L of PBS (containing 3% FBS, PBSF). The washed cells were resuspended in 100 *μ*L of PBSF for the analysis of surface markers of MSCs. Each cell sample was incubated with 5 *μ*L (10 *μ*g/*μ*L) of antihuman PE-CD44, APC-CD73, FITC-CD90, PE-CD105, PE-CD105, PE-CD59, HLA-A,B,C, PE-CD45, PE-CD14, PE-CD34, PE-CD11b, PE-CD19, and PE-HLA-DR antibodies for 1 h on ice, and isotype control antibodies were used in parallel. Unbound antibodies were washed off with PBSF, and subsequently, the cells were resuspended in 500 *μ*L of PBSF.

### 2.4. Differential Potency Evaluation of Bone Marrow-Derived MSCs

#### 2.4.1. Adipogenic Differentiation

The bone marrow-derived MSCs were seeded onto 24-well plates and cultured at a density of 8 × 10^4^ cells per well for 12 h. Subsequently, the cells were cultured in adipogenic differentiation medium (Gibco BRL, Grand Island, NY, USA) for 7 days [26]. The medium was refreshed every 3 days. The cells were stained using filtered Oil Red O (0.2% Oil Red O in 60% isopropanol, *v*/*v*) for 15 min and washed 3 times with PBS after fixation in 4% methanol. The adipogenic differentiation was confirmed as the appearance of cellular accumulation of neutral lipid vacuoles was stained red with Oil Red O (Sigma, St Louis, USA).

#### 2.4.2. Osteogenic Differentiation

The bone marrow-derived MSCs were seeded onto 24-well plates and cultured at the density of 4 × 10^4^ cells per well for 12 h. Subsequently, the culture medium was replaced with osteogenic differentiation medium (Gibco BRL, Grand Island, NY, USA) and further cultured for 21 days. The medium was refreshed every three days. The cells were stained with fresh 0.5% alizarin red solution and washed 3 times with PBS, followed by fixation with 4% methanol. The osteogenic differentiation was confirmed as the appearance of alizarin red staining.

#### 2.4.3. Chondrogenic Differentiation

The bone marrow-derived MSCs were collected in 15 mL centrifuge tubes containing approximately 2 × 10^5^ per tube and subsequently cultured in chondrogenic differentiation medium (Gibco BRL, Grand Island, NY, USA). The medium was refreshed every three days. After 21 days of differentiation induction, the chondroid pellets were generated and washed with PBS and fixed in 4% paraformaldehyde, embedded with optimum cutting temperature (OCT) embedding material (Leica, Wetzlar, Germany). The pellets were sectioned using a freezing microtome, and subsequently, sulfated proteoglycans were visualized by staining with 1% toluidine blue (Merck, Darmstadt, Germany) for 10 min [27]. These slices were washed 3 times with PBS and photographed under an inverted microscope. The differentiation was confirmed as the appearance of alcian blue staining.

### 2.5. Vitrification of MSCs

The bone marrow-derived MSCs from the three donors were harvested at passage 5 for the vitrification assay when the cells reached 80% confluency. The cell suspension was divided into three equal aliquots at a density of 2 × 10^6^ cells/mL. One of the aliquots without cryopreservation was subcultured in fresh medium for 24 h, and cell viability, immunophenotype surface markers, proliferation, and metabolic activity were subsequently examined as a nonvitrified control (VC). The other two aliquots were vitrified using either DMSO (Sigma, St Louis, USA) (VD) or EG (Sigma, St Louis, USA) (VE) as a penetrative cryoprotectant, respectively. The vitrification protocol contained a two-step exposure to equilibration and vitrification solutions, respectively [23]. The equilibration solution contained 2.8 M DMSO or EG, and the vitrification solution comprised 5.6 M DMSO or EG, 18% Ficoll 70 (Sigma, St Louis, USA), and 0.3 M sucrose (Sigma, St Louis, USA). All solutions were based on a PBS solution containing 20% FBS. Briefly, a total of 1 × 10^6^ MSCs were suspended in 50 *μ*L of equilibration solution for 5 min and subsequently mixed with 500 *μ*L of vitrification solution for 40 s (step 1). The suspended MSCs in vitrification solution were immediately transferred to 1 mL cryovials (Corning, NY, USA) and directly frozen in liquid nitrogen (step 2). After storing in liquid nitrogen for 24 h, the cells were rapidly warmed by immersing the cryovial in a 37°C water bath for 5 min [28, 29]. The cells were sequentially washed in a PBS containing 20% FBS supplemented with 0.5, 0.25, and 0 M sucrose for 3 min each. Finally, the MSCs were resuspended and cultured in DMEM medium supplemented with 20% FBS for 24 h. Cells cultured using the same conditions without vitrification served as controls. The morphology, cell viability, immunophenotype of cell surface markers, proliferation and metabolic activity, and gene expression of the MSCs from the VC, VD, and VE groups were subsequently evaluated as described in the following assays.

### 2.6. Cell Viability Assay

The percentages of viable cells from vitrified (VD and VE, resp.) and control (VC) groups were assessed using a trypan blue dye exclusion assay at 1 : 1 dilution (0.4% trypan blue in PBS). The stained and total numbers of cells (approximately 100 cells) were counted using a hemocytometer under a microscope. This assessment was repeated three times.

### 2.7. Proliferation Ability and Metabolic Activity

The proliferation ability and metabolic activity of MSCs from vitrified (VD and VE) and control (VC) groups were determined using an MTS (3-(4,5-dimethylthiazol-2-yl)-5-(3-carboxymethoxyphenyl)-(4-sulfophenyl)-2H-tetrazolium) assay. Briefly, 200 *μ*L of cell suspension was seeded onto a 96-well plate at a density of 10^4^ cells/well. Subsequently, 20 *μ*L of CellTiter 96 AQueous One Solution Reagent (Promega, Beijing, China) was added to each well and incubated for 2 h. The quantity of formazan product is directly proportional to the number of living cells in culture. The colored formazan was measured at 490 nm in a 96-well microplate reader at 0, 12, 24, 48, and 72 h [30]. This assessment was repeated three times.

### 2.8. Transcriptome Profiles of Control MSCs and Vitrified MSCs in DMSO and EG

The cells from the VC, VD, and VE groups were collected and resuspended in Trizol Reagent (Takara, Dalian, China) and stored in a −80°C freezer, respectively. The total RNA was extracted from each sample. The RNA quality of each group was assessed using agarose gel electrophoresis, and the RNA purity was assessed using the NanoPhotometer® spectrophotometer (IMPLEN, CA, USA). The concentration of RNA was measured using the Qubit® RNA Assay Kit in Qubit 2.0 Fluorometer (Life Technologies, CA, USA). RNA integrity was assessed using the RNA Nano 6000 Assay Kit of the Bioanalyzer 2100 system (Agilent Technologies, CA, USA). Finally, RNA-seq procedures were performed at Novogene Co. (Beijing, China). A total amount of 3 *μ*g RNA per sample was used as an input material for the RNA sample preparations. Sequencing libraries were generated using the NEBNext® Ultra™ RNA Library Prep Kit for Illumina® (NEB, USA) according to the manufacturer's instructions, and index codes were added to attribute sequences to each sample. Briefly, mRNA was purified from total RNA using poly-T oligo-attached magnetic beads. Fragmentation was performed using divalent cations under an elevated temperature in NEBNext First Strand Synthesis Reaction Buffer (5×). First-strand cDNA was synthesized using random hexamer primer and M-MuLV Reverse Transcriptase (RNase H). Second-strand cDNA synthesis was subsequently performed using DNA polymerase I and RNase H. The remaining overhangs were converted into blunt ends via exonuclease/polymerase activities. After the adenylation of 3′ ends of DNA fragments, NEBNext Adaptors with hairpin loop structure were ligated to prepare for hybridization. To select cDNA fragments of preferential 150~200 bp in length, the library fragments were purified using the AMPure XP system (Beckman Coulter, Beverly, USA). Subsequently, 3 *μ*L of USER enzyme (NEB, USA) was used with size-selected, adaptor-ligated cDNA at 37°C for 15 min followed by 5 min at 95°C prior to PCR. Subsequently, PCR was performed using Phusion High-Fidelity DNA polymerase, universal PCR primers, and Index (X) Primer. Finally, the PCR products were purified (AMPure XP system), and library quality was assessed on the Agilent Bioanalyzer 2100 system. The clustering of the index-coded samples was performed on the cBot Cluster Generation System using TruSeq PE Cluster Kit v3-cBot-HS (Illumina) according to the manufacturer's instructions. After cluster generation, the library preparations were sequenced on an Illumina Hiseq platform and 125 bp/150 bp paired-end reads were generated.

### 2.9. Differential Gene Expression Validated Using qRT-PCR

Several selected differentially expressed genes (Table 1) among the VC, VD, and VE groups detected in RNA-Seq were further validated using qRT-PCR. Briefly, total RNA was extracted from the MSCs from the VC, VD, and VE groups using Trizol Reagent (Takara, Dalian, China). The RNA was first separated into an aqueous phase using chloroform, subsequently precipitated with isopropanol, rinsed with 75% ethanol, and finally solubilized in sterile DEPC water. Complementary DNA (cDNA) was subsequently synthesized using a PrimeScript RT reagent kit (Takara, Dalian, China) according to the manufacturer's instructions. Highly purified gene-specific primers (Table 1), including the housekeeping gene glyceraldehyde-3-phosphate dehydrogenase (GAPDH), were commercially synthesized (Shenggong, Shanghai, China). Quantification of the cDNA of specific genes was performed with a Bio-Rad CXF real-time PCR system. All experiments were performed in triplicate, and the data were analyzed using 2^−△Ct^ procedures.

### 2.10. Statistical Analysis

The results from these experiments are presented as the means ± SD. The statistical significance of the cell viability, proliferation, and metabolic activities between the VC, VD, and VE groups were determined using SPSS 17.0 software with one-way analysis of variance (ANOVA) and Fisher's protected least significant difference test. A *P* value less than 0.05 was considered statistically significant. Differential gene expression analysis of the VC, VD, and VE groups was performed using the DESeq R package (1.18.0). DESeq provides statistical routines for determining differential expression in digital gene expression data using a model based on the negative binomial distribution. The resulting *P* values were adjusted using Benjamini and Hochberg's approach for controlling the false discovery rate. Genes with an adjusted *P* value < 0.05 according to DESeq were assigned as differentially expressed. Gene ontology (GO) enrichment analysis of differentially expressed genes was implemented using the GOseq R package, in which gene length bias was corrected. GO terms with corrected *P* values less than 0.05 were considered significantly enriched with differentially expressed genes.

## 3. Results

### 3.1. Morphology, Surface Marker Profiles, and Differentiation Potency of Bone Marrow-Derived MSCs

**Figure 1:**
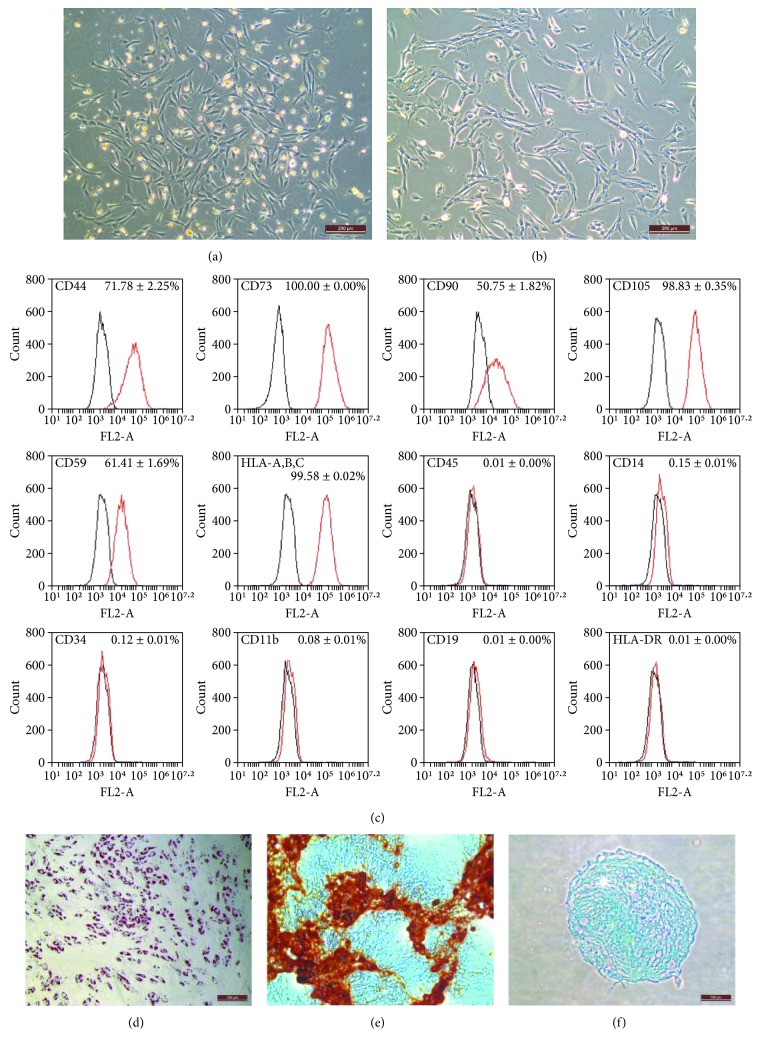
Adherent, fibroblast-like morphology of MSCs at passage 0 (a) and passage 3 (b). Scale bars: 200 *μ*m. (c) Surface marker expression on bone marrow-derived MSCs at passage 3 analyzed using flow cytometry. Black lines represent isotype control. (d–f) Differentiation potency of MSCs at passage 3. (d) Adipogenic differentiation (oil red staining, ×200). (e) Osteogenic differentiation (alizarin red staining, ×200). (f) Chondrogenic differentiation (alcian blue staining, ×200). Scale bars: (d) and (e) were 200 *μ*m, and (f) was 100 *μ*m.

During primary culture, the MSCs derived from macaque bone marrow grew adhesively in plastic dishes in a scattered manner. The cells that formed colonies and appeared heterogeneously were referred to as passage 0. The MSCs started to appear homogenous fibroblast-like, elongated, and spindle-shaped with single nuclear features following subsequent culture (Figure 1(a)). The MSC colonies at passage 0 were extended to passage 3 with progressive subculture, and the morphology of MSCs at passage 3 also showed heterogeneous and fibroblast-like shapes (Figure 1(b)). The identification of MSCs was performed at passage 3 as shown in the previous studies [31, 32]. The surface marker profiles of the bone marrow-derived MSCs were analyzed at passage 3 using flow cytometry. The results indicated that the cells positively expressed high levels of CD44, CD73, CD90, CD105, CD59, and HLA-A,B,C but negatively expressed CD45, CD14, CD34, CD11b, CD19, and HLA-DR (Figure 1(c)). The cells from adipogenic differentiation of MSCs formed numerous neutral lipid droplets in the cytoplasm as identified using Oil Red O staining (Figure 1(d)). The cells from osteogenic differentiation of MSCs showed mineral accumulation and bone nodule formation as identified using alizarin red staining (Figure 1(e)). The cells from chondrogenic differentiation of MSCs were identified using alcian blue staining (Figure 1(f)).

### 3.2. Vitrification of MSCs at Passage 5

#### 3.2.1. Compared to Cells Vitrified Using EG, Vitrified MSCs Using DMSO Showed Lower Cell Viability, Proliferation, and Metabolic Activity

**Figure 2:**
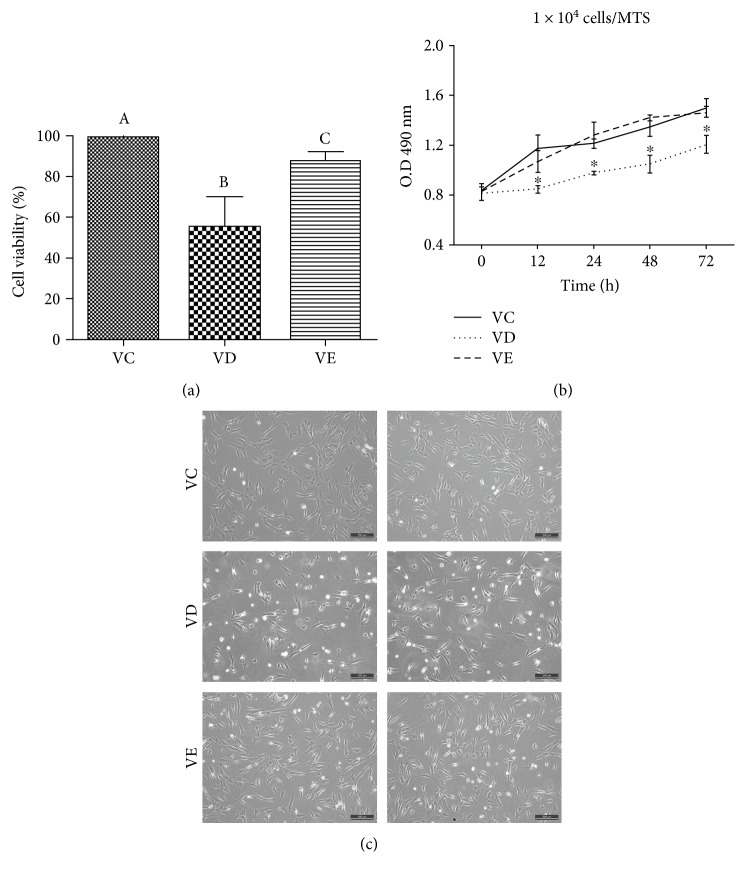
(a) Comparison of the cell viability between nonvitrified control (VC) and vitrified MSCs (VD and VE). Different superscripts indicate significant differences (*P* < 0.05). (b) The proliferation ability and metabolic activity of MSCs of the VC, VD, and VE groups after warming and 24 h of culture. ∗ represents significant differences among the three groups (*P* < 0.05). (c) The morphology of control MSCs (VC) and vitrified MSCs after being thawed and cultured for 24 h (VD and VE). Scale bars: 200 *μ*m.

Vitrification significantly decreased the viability of MSCs using either DMSO (VD, 54.93 ± 13.07%) or EG (VE, 87.31 ± 4.36%) as penetrating cryoprotectants compared to that of the nonvitrified control group (VC, 98.83 ± 1.03%), and compared to EG, DMSO showed less cryoprotection of cells (Figure 2(a), *P* < 0.05). The overall cell metabolic activities of VC, VD, and VE at different time points after a 24 h culture are shown in Figure 2(b). The results showed that the proliferation and metabolic activity of the cells of the VD group were significantly lower than those of the VC and VE groups at 12, 24, 48, and 72 h (Figure 2(b), *P* < 0.01). However, no differences were observed between the cells of VC and VE at any time point (Figure 2(b), *P* > 0.05). The morphology of warmed cells after a 24 h subculture is shown in Figure 2(c). No obvious morphological changes were observed among the three groups, except that the confluency of VD was sparse compared to the VC and VE groups.

#### 3.2.2. Expression of Surface Markers Was Not Affected by Vitrification

**Figure 3:**
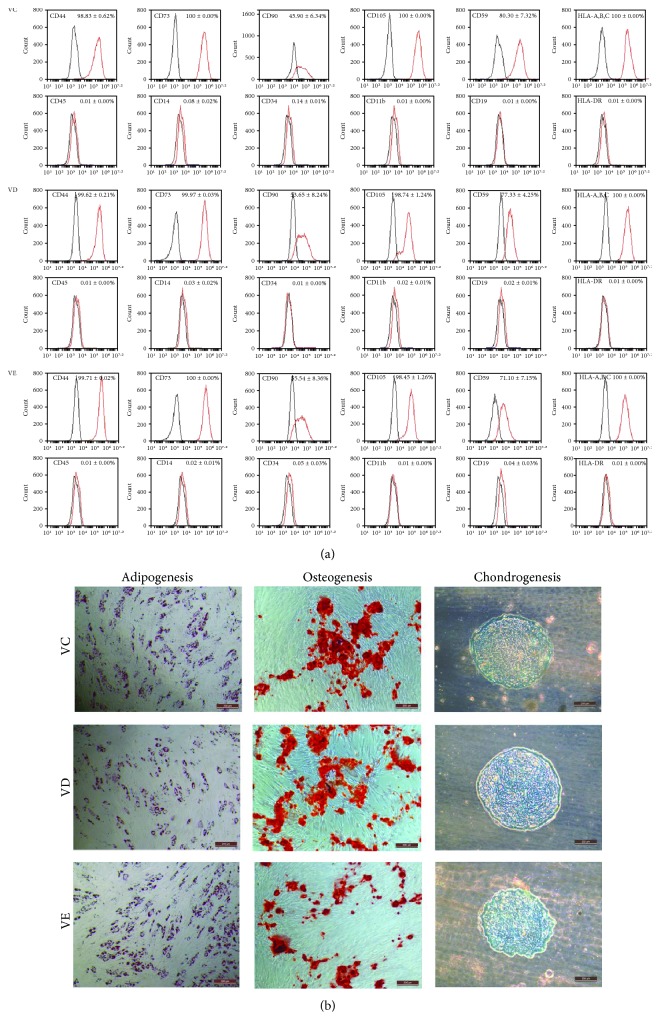
(a) Surface marker expression on vitrified MSCs from the VD, VE, and VC groups analyzed by flow cytometry. Black lines represent isotype control. (b) Differentiation potency of MSCs from the VC, VD, and VE groups. The cells with adipogenic induction show lipid droplets identified using oil red staining; the cells with osteogenic induction show the aggregation of micronodules or calcium deposits identified using alizarin red staining; the cells with chondrogenic induction show vacuolized cells identified using alcian blue stain. Scale bars: 200 *μ*m.

After warming and following 24 h of subculture, the surface marker profiles of vitrified MSCs from the VD and VE groups were compared to those from the control cells (VC). The MSCs of all groups showed a positive immunophenotype of CD44, CD73, CD105, CD59, HLA-A,B,C, and CD90 and a negative immunophenotype of CD45, CD14, CD34, CD11b, CD19, and HLA-DR (Figure 3(a)).

#### 3.2.3. Differentiation Potency Was Not Affected by Vitrification

Similar to the cells in the VC group, the vitrified MSCs from either the VD or VE group differentiated into adipocytes, osteocytes, and chondrocytes (Figure 3(b)). After adipogenic induction, numerous neutral lipid droplets stained with Oil Red were observed in the cytoplasm of the cells from the VC, VD, and VE groups (negative controls of VD and VE were showed in Figure S1 available online at https://doi.org/10.1155/2017/3893691). After osteogenic induction, the vitrified MSCs (VD and VE) and control cells (VC) presented an aggregation of micronodules or calcium deposits that was stained with alizarin red. The chondrogenic differentiation of warmed MSCs and control cells could be observed using alcian blue stain.

### 3.3. Transcriptome Profiles of MSCs Were Changed in Large Scale after Vitrification Using Either DMSO Or EG

**Figure 4:**
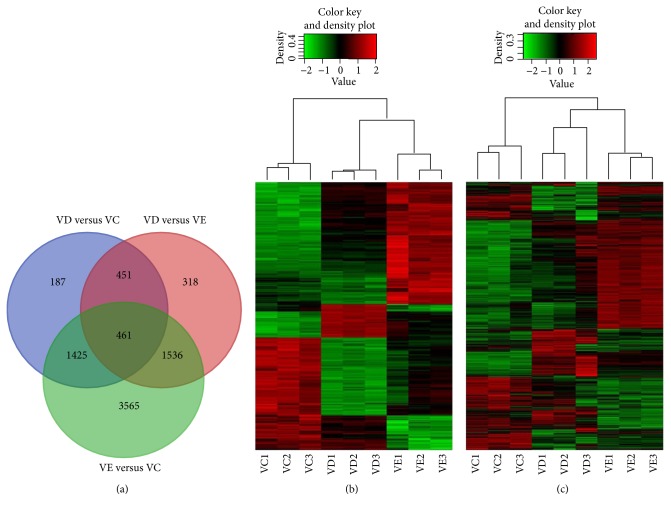
Gene expression pattern of the MSCs from the nonvitrified control (VC) and vitrified (VD and VE) groups. (a) Venn diagrams showing the differences in gene expression among the three groups. (b) Heat maps indicating the intensity of the total differentially expressed genes (7943 genes) shown in Figure 4(a) of the three groups. (c) Heat map presenting 461 genes differentially expressed in the three groups simultaneously, presenting an intersection among VC, VD, and VE. Red denotes upregulated genes, and green denotes downregulated genes.

**Figure 5:**
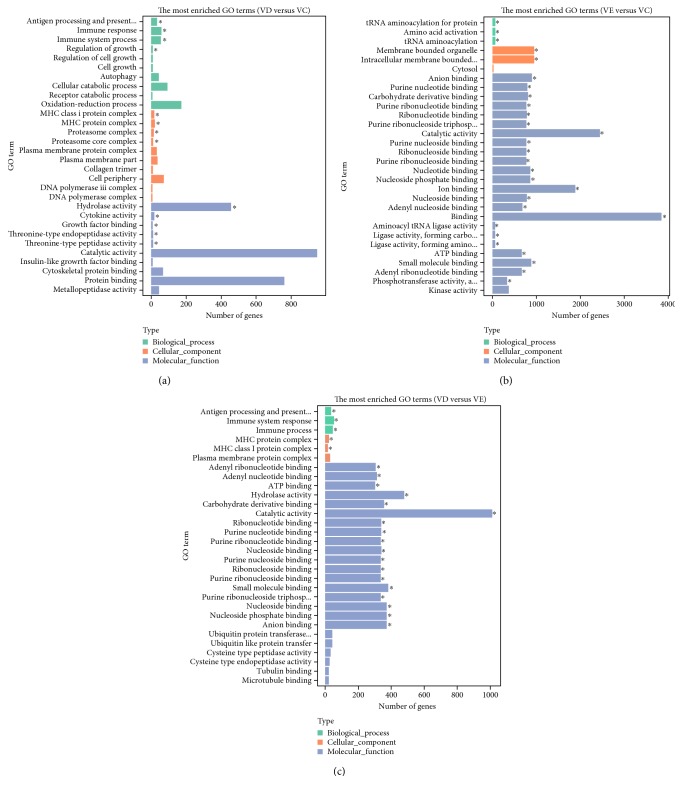
The enrichment analysis of Gene Ontology. Enriched terms are colored in green, orange, or blue, corresponding to biological process, cellular component, or molecular function, respectively. The most enriched GO terms: (a) VD versus VC, (b) VE versus VC, and (c) VD versus VE. ∗ represents *P* < 0.05.

The number of significantly modulated genes among MSCs from the VC, VD, and VE groups is summarized in Table 2. The results showed that after vitrification and warming for 24 hours, the MSCs cryopreserved using either DMSO or EG (VD or VE group) presented a large number of changed transcripts compared to those from the nonvitrified control (VC group). The results of the Venn diagram analysis of gene regulation in the three groups are shown in Figure 4(a). The result showed 2524 differentially expressed genes between VD and VC, 6987 differentially expressed genes between VE and VC, and 2766 differentially expressed genes between VD and VE. Compared to the transcriptome profiles of control MSCs, the vitrified MSCs from VE showed many more effects on the up- and downregulation of gene expression than those cells from VD. As shown in the clustering analysis according to the Venn diagram in Figure 4(a), a total of 7943 differentially expressed genes were discovered after vitrification and warming among the MSCs from the VC, VD, and VE groups. The heat map presents 7943 differentially expressed genes among the three groups (Figure 4(b)). According to the Venn diagram shown in Figure 4(a), 461 genes were differentially expressed in the three groups simultaneously and presented an intersection among VC, VD, and VE. The heat map of the 461 genes differentially expressed among the three groups is presented in Figure 4(c). The enrichment analysis of Gene Ontology (GO) between MSCs of VC, VD, and VE is presented in Figure 5. The graph displays the distribution of the biological terms in the ontology of GO terms that was presented in biological process, cellular component, and molecular function.

### 3.4. Differential Gene Expression Validated Using qRT-PCR

**Figure 6:**
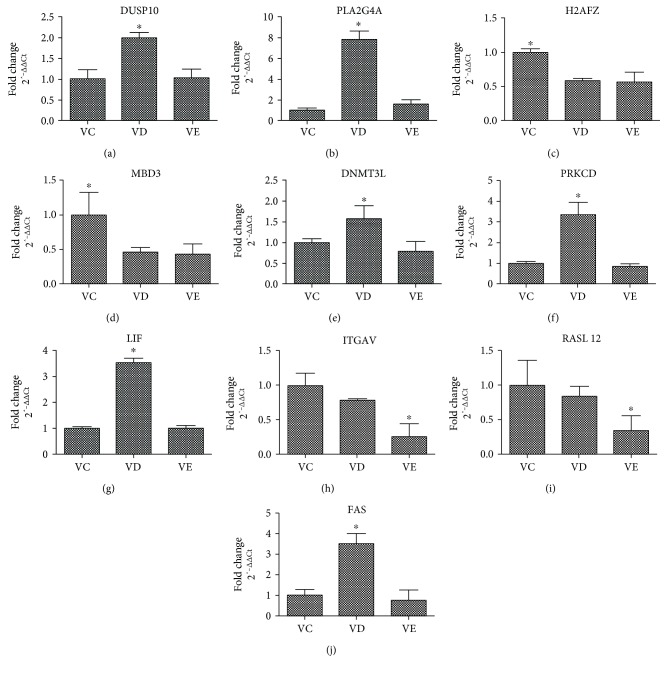
The differential expression of the selected 10 genes validated using qRT-PCR. (a) DUSP10: Dual specificity phosphatase 10, immune pathway-related gene. (b) PLA2G4A: phospholipase A2 group IVA, cell signaling-related gene. (c) H2AFZ: H2A histone family member Z, (d) MBD3: methyl-CpG binding domain protein 3, and (e) DNMT3L: DNA (cytosine-5-)-methyltransferase 3-like are epigenetic regulation-related genes. (f) PRKCD: protein kinase C delta, cell signaling-related gene. (g) LIF: leukemia inhibitory factor, cell differentiation-related gene. (h) ITGAV: integrin subunit alpha V, cell adhesion- and signal transduction-related gene. (i) RASL 12: RAS-like family 12, metabolic pathway-related gene. (j) FAS: Fas cell surface death receptor, cell apoptosis-related gene. ∗ represents significant differences among the three groups (*P* < 0.05).

The differentially expressed genes were separated into several categories according to their functions, including immune pathway, cell signaling, epigenetic regulation, cell differentiation, cell adhesion and signal transduction, metabolic pathway, and cell apoptosis. The 10 selected genes were determined using qRT-PCR to confirm the results of the transcriptome profiles of MSCs according to original analysis results of RNA-Seq, including the up- or downregulated differentially expressed genes and corresponding multiples. The expression levels of the 10 genes among MSCs from the control (VC) and vitrified groups (VD and VE) are summarized in Figure 6. Genes encoding proteins related to immune pathway (DUSP10), cell signaling (PLA2G4A, PRKCD), epigenetic regulation (DNMT3L, H2AFZ, MBD3), cell differentiation (LIF), cell adhesion and signal transduction (ITGAV), metabolic pathway (RASL 12), and cell apoptosis (FAS) were differentially expressed among the three groups, consistent with the results determined using RNA-Seq. Based on these results, the effects of EG and DMSO on the biological characteristics of MSCs after vitrification and warming were confirmed.

## 4. Discussion

In the present study, we used rhesus macaque as a model to establish bone marrow-derived mesenchymal stem cell lines and investigate the effects of vitrification using two common penetrating cryoprotectants on the self-renewing capacity and *in vitro* differentiation and global gene expression of MSCs. The MSCs derived from the bone marrow of macaques presented features of heterogeneous, fibroblast-like, and spindle-shaped morphology, consistent with the morphology of the bone marrow-derived MSCs as previously reported [33]. The cells expressed typical positive and negative surface markers of MSCs [33]. These results indicated that positive and negative surface molecules were consistently expressed in humans and macaques. The tri-lineage differentiation of MSCs into adipocytes, osteocytes, and chondrocytes under respective inductive conditions were considered as the main process for identifying MSCs with the functional capacity for cell therapy [33]. The isolated macaque MSCs in the present study are consistent with the definition of monkey MSCs, with a typical morphology, cell surface marker expression, and tri-lineage differentiation potency as previously described [34].

Cryopreservation plays an important role in maintaining cell function for tissue engineering, cell transplantation, pharmacological testing, and future therapeutic indications [35]. Traditionally, DMSO is used as cryoprotectant agent in both conventional slow freezing and vitrification protocols for MSCs [36]. Due to side effects, such as nausea, chills, hypotension, and cardiac arrhythmia, that have been reported in humans during the infusion of cryopreserved stem cells, efforts to develop a cryopreservation protocol with low levels of DMSO or DMSO-free conditions are needed to avoid the toxic effects. Moon and colleagues preserved human amnion-derived MSCs using a DMSO-free vitrification protocol with a 2-step procedure. MSCs were vitrified in a solution containing 40% ethylene glycol, 18% Ficoll, 0.3 M sucrose, and 20% FBS for 40 s. The result showed 84.3 ± 3.2% postthaw cell viability [23]. Massood and colleagues vitrified human umbilical cord Wharton's jelly-derived MSCs using Moon's protocol, and their results showed 95.54 ± 2.30% postthaw viability and the retention of surface antigens and tri-lineage differentiation [37]. However, to date, the differences in cryoprotective roles between DMSO and EG on vitrification of MSCs have not been studied. The present study compared the two different cryoprotectants (DMSO and EG) on the cryosurvival, proliferation, and differentiation potency of the macaque bone marrow-derived MSCs. The results showed that the postwarmed viability of MSCs vitrified using DMSO and EG was 54.93 ± 13.07% and 87.31 ± 4.36%, respectively. The viability of MSCs vitrified with EG in the present study was at the same level compared to that of human amnion-derived and fetal liver-derived MSCs vitrified with EG using a similar protocol in the previous studies [23, 38], and compared to previous studies, a low level of EG was used in the present study (31.33% versus 40%, *v*/*v*). The optimal concentration of cryoprotectant for primate MSC vitrification should be further explored and optimized.

In the present study, the viability of MSCs vitrified using DMSO was significantly lower than that using EG, and the cells of VD groups showed sparse confluency after culture for 24 h and a low proliferation metabolic activity compared to MSCs of the VE and control groups. During vitrification, a high concentration of cryoprotectant was used, which may lead to cytotoxic effects. Thus, DMSO-free protocols for either traditional slow freezing or vitrification have been developed to optimize stem cell cryopreservation [38, 39]. Comparative studies investigating the effects of DMSO and EG on the cryosurvival of induced pluripotent stem cells and Wharton's jelly tissue using a slow cooling method demonstrated that EG had better cryoprotection than DMSO [19, 24]. Vitrifying MSCs with high levels of EG instead of DMSO also achieved desired cell survivability [23, 38]. Compared to DMSO, EG is less toxic to macaque sperm, mouse, and human embryos [20, 40, 41], suggesting that EG might be more appropriate for macaque MSC vitrification. However, there are no studies that directly compare the effect of EG and DMSO on the vitrification of primate MSCs. In the present study, the viability of macaque MSCs vitrified with DMSO was lower than that of cells vitrified with EG. This result indicates that EG was less toxic and provided more cryoprotection for macaque MSC vitrification than DMSO. DMSO is intrinsically toxic to cells and can activate apoptosis pathways and cause mitochondrial membrane damage and posttransplantation complications [14, 17], which might be one of the reasons that the MSCs preserved with DMSO showed low cryosurvival rate and metabolic activities. In addition, the permeability coefficient of EG and DMSO to macaque bone marrow-derived MSCs is unknown. Study on the permeability of sperm membrane indicated that EG might be the most appropriate CPA for rhesus sperm freezing due to its high permeability coefficient [42]. We propose that the macaque MSC membrane is more permeable to EG than DMSO. EG might cause less osmotic stress during the addition of the cryoprotectant prior to vitrification and the removal of cryoprotectant after warming and therefore could provide sufficient cryoprotection and result in fewer cell injuries.

Recent studies have demonstrated that, although the basic characteristics of plasticity and multipotency of MSCs were not altered after cryopreservation with DMSO by traditional slow freezing, the expression of apoptosis-related genes, such as BAC, BCL-2, BAX, P53, and P21, was affected [7, 19]. However, to our knowledge, the high-throughput gene expression profiles of MSCs after cryopreservation through either traditional slow freezing or vitrification methods have not been documented. Nevertheless, cryopreservation by either slow cooling or vitrification has a profound effect on gene expression, as primarily demonstrated in reproductive cells. For example, vitrification affected the expression of apoptosis-related genes of mouse follicles, zygotes, and embryos [43–45]; both slow freezing and vitrification differentially modified the gene expression profile of human metaphase II oocytes [46]. Studies have revealed that exposure to DMSO, even at a low concentration (0.02–1.0%), impacted the epigenetic profile of embryonic stem cells and embryoid bodies and resulted in the upregulation of DNA methyltransferase expression and alterations of genome-wide DNA methylation profiles with phenotypic changes [47]. Similarly, the exposure of endothelial cells to high levels of EG resulted in changes of gene expression profiling as demonstrated by the whole genome microarrays [48]. In the present study, vitrification using either DMSO or EG resulted in changes in a large number of transcripts of macaque bone marrow-derived MSCs, indicating that vitrification had a significant impact on the whole genome expression. Ethylene glycol is a less toxic cryoprotectant than DMSO. The results showed that MSCs vitrified with EG showed higher cryosurvival and proliferation than those by DMSO, consistent with the low toxicity of EG. However, EG impacted the expression of many more genes than DMSO in the present study. The epigenetic profile of MSCs vitrified using DMSO or EG was also affected, and the upregulation of DNA methyltransferase expression in MSCs vitrified with EG (DNMT1 and DNMT3A) and DMSO (DNMT3L) was observed. The results are similar to those of a previous study reporting that the global gene expression and epigenetic profile of embryonic stem cells were affected by exposure to DMSO [46, 47]. Notably, in the present study, MSCs were vitrified using the same protocol, but different CPAs (DMSO and EG). The numerous changes in the global gene profile resulted from CPAs and vitrification. However, differences in the global gene expression between fresh MSCs and the DMSO or EG group might reflect the cooling/warming process. In contrast, the effect of cooling/warming on global gene expression between the MSCs in the DMSO and EG groups could be excluded. The critical concentration of CPA and the effect of cooling/warming cycle on the global gene profile are unknown, and whether the effects on the global gene expression are permanent or temporary was not determined in the present study. Further experiments are needed to clarify these questions. Moreover, in the present study, although vitrification using either DMSO or EG resulted in changes in a large number of transcripts of MSCs, and low viability, proliferating ability, and metabolic activity of MSCs vitrified using DMSO was observed, the morphology, surface immunophenotypes, and tri-lineage differentiation potency of MSCs were not affected. Recently, a spectacular difference between mRNA and protein was revealed by measuring genome-wide transcript and protein expression in mouse liver, suggesting that modulating the level of protein from mRNA rather than depending on a simple central dogma is complex [49]. In addition, studies on ovarian cancer revealed that proteins show function through protein modification and interactions with other proteins rather than high or low expression levels of mRNA and protein revealed through proteogenomics [50]. This phenomenon may explain why the affected gene expression of MSCs was changed, but the biological characteristics of morphology, surface immunophenotypes, and differentiation potency were not affected in the present study.

In conclusion, vitrification using either DMSO or EG did not affect the morphology, surface markers, and differentiation of MSCs. However, MSCs vitrified using DMSO showed poor cell viability and proliferation ability compared to those vitrified using EG. The vitrification of MSCs using either DMSO or EG leads to changes in a large number of transcripts compared to those of control cells. This report is the first to show the different effects of DMSO and EG on the global gene expression and impact of EG on the epigenetic profile of stem cells. These results will be beneficial to understanding the biological process involved in the vitrification of MSCs and contribute to improved cryopreservation protocols that maintain transcriptomic identity with high cryosurvival for preclinical research and clinical long-term storage.

## Supplementary Material

Figure S1. The MSCs vitrified with DMSO (a) and EG (b) were cultured in DMEM medium supplemented with 10% FBS for 7 days without differentiation induction as negative controls. No adipogenic differentiation was observed evaluated with oil red staining.

## Figures and Tables

**Table 1:** The primer information for qRT-PCR.

Gene	NCBI ID	Primer sequences	PCR production (bp)
FAS	NM_001032933.2	S: 5′ ACACTCACCAGCAACACCAA 3′ A: 5′ TTCACTGACACCATTCTTTCG 3′	291
PRKCD	XM_005547391.2	S: 5′ CACAGCAAGGGCATCATTTAC 3′ A: 5′ AGACCACCAGTCCACCGAGA 3′	207
RLA2G4A	XM_015121786.1	S: 5′ AAACTCTAGGGACCGCAACA 3′ A: 5′ GCTACCACAGGCACATCACG 3′	274
RASL	NM_001265994.1	S: 5′ GACCACCAGCCTGTCCACC 3′ A: 5′ CCAAACCTGCCTGCCAAA 3′	281
H2AFZ	NM_001193550.1	S: 5′ TACTTGAACTGGCAGGAAATG 3′ A: 5′ ATGACACCACCACCAGCAAT 3′	163
ITGAV	NM_001265953.1	S: 5′ CGGGACTCCTGCTACCTCTG 3′ A: 5′ CTGGGTCGTGTTTGCTTTGG 3′	170
LIF	XM_015150132.1	S: 5′ CAGTGCCAATGCCCTCTTTAT 3′ A: 5′ CACGGCGATGGTCTCCTTAT 3′	152
DUSP10	NM_001257695.2	S: 5′ TTTAGACGACAGGGTAGTAGT 3′ A: 5′ GCAGCAATGGCTTGGGTTT 3′	284
MBD3	NM_001194043.1	S: 5′ ATGGAGCGGAAGAGGTGG 3′ A: 5′ GGTTGGAGGAGTCGTAGCG 3′	180
DNMT3L	XM_015132779.1	S: 5′ CCCTGTGGTCCCTGGTTTC 3′ A: 5′ GCCCTCCAAGGCTGTCC 3′	118
GAPDH	NM_001195426.1	S: 5′ ACGGATTTGGTCGTATTGG 3′ A: 5′ GCTCCTGGAAGATGGTGAT 3′	150

**Table 2:** Number of modulated genes of MSCs vitrified with DMSO (VD) and EG (VE) compared to those of nonvitrified MSCs from the control (VC).

Comparisons	Differential express gene number		
	Total	Upregulated	Downregulated
VD versus VC	2524	1205	1319
VE versus VC	6987	3433	3554
VD versus VE	2766	1370	1396
