# Supplementary material for: Vitrification of Rhesus Macaque Mesenchymal Stem Cells and the Effects on Global Gene Expression
Source: Stem Cells Int. 2017 Oct 24;2017:3893691. doi: 10.1155/2017/3893691 (PMC5674518; doi:10.1155/2017/3893691)
Supplement: Supplementary file 1 — Figure S1. The MSCs vitrified with DMSO (a) and EG (b) were cultured in DMEM medium supplemented with 10% FBS for 7 days without differentiation induction as negative controls. No adipogenic differentiation was observed evaluated with oil red staining. [file 3893691.f1.pdf]

Supplementary materials

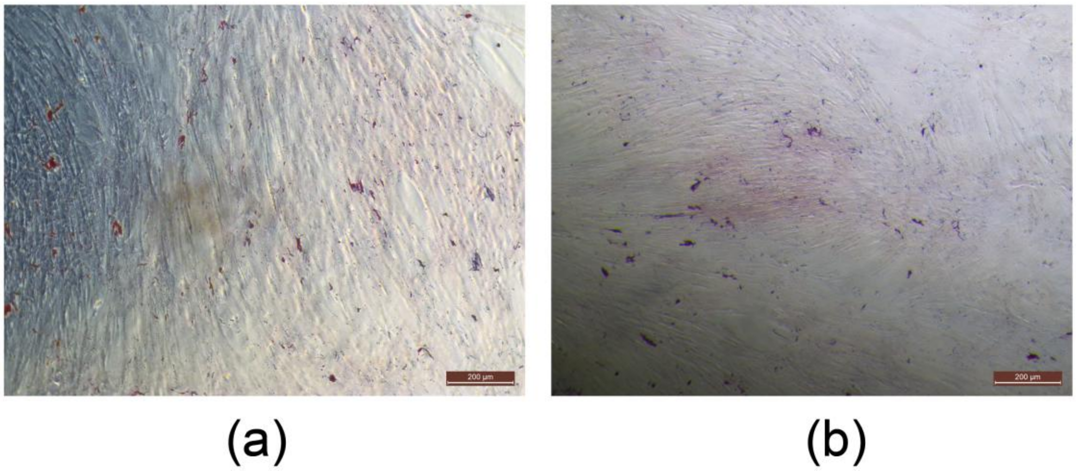

Figure S1. The MSCs vitrified with DMSO (a) and EG (b) were cultured in DMEM medium supplemented with 10% FBS for 7 days without differentiation induction as negative controls. No adipogenic differentiation was observed evaluated with oil red staining.
